# The Neutrophil-to-Lymphocyte ratio as a marker of recovery status in patients with severe dental infection

**DOI:** 10.4317/medoral.21915

**Published:** 2017-06-18

**Authors:** Fatma Dogruel, Zeynep-Burcin Gonen, Dilek Gunay-Canpolat, Gokmen Zararsiz, Alper Alkan

**Affiliations:** 1 MD, Department of Oral and Maxillofacial Surgery, Faculty of Dentistry, Erciyes University, Assistant Professor in Internal Medicine, Kayseri, Turkey; 2 DDs,PhD, Genome and Stem Cell Center and Department of Oral and Maxillofacial Surgery, Faculty of Dentistry, Erciyes University, Assistant Professor, Kayseri, Turkey; 3 MD, Department of Oral and Maxillofacial Surgery, Faculty of Dentistry, Erciyes University, Assistant Professor in Anesthesiology, Kayseri, Turkey; 4 PhD, Department of Statistics, Erciyes University Medical School, Kayseri, Assistant Professor, Kayseri, Turkey; 5 Turcosa Analytics Solutions Ltd. Co, Erciyes Teknopark, Kayseri, Turkey; 6 DDs, PhD, Department of Oral and Maxillofacial Surgery, Faculty of Dentistry, Bezmialem University, Professor, İstanbul, Turkey

## Abstract

**Background:**

The aim of the study was to assess the value of pretreatment neutrophil/lymphocyte (N/L) ratio and mean platelet volume (MPV) and the correlation between these markers with progression in patients with severe odontogenic infection.

**Material and Methods:**

A cohort of 100 patients with severe odontogenic infection were divided into 2 groups according to their length of hospital stay. The N/L ratio and MPV was measured in all patients. The correlation in all patients between preoperative fever, preoperative antibiotic doses, postoperative antibiotic doses, total antibiotic doses and hospital stay with N/L ratio and MPV were analyzed. The Youden index was used to identify the optimal cut-off value.

**Results:**

There were positive and statistically significant correlations between N/L ratio and prolonged hospital stay and postoperative antibiotic doses and total antibiotic doses. The optimum cut –off level of N/L ratio was 5.19 according to ROC analysis. However, there was no correlation between MPV and any of these parameters.

**Conclusions:**

N/L ratio may be used as a prognostic marker for patients with odontogenic infections. These patients may need a higher dose of antibiotics and stay more than 1 day in hospital for the treatment of odontogenic infection when the N/L ratio is detected to be more than 5.19.

** Key words:**Neutrophil/lymphocyte ratio, mean platelet volume, odontogenic infection.

## Introduction

Patients with odontogenic infections need to be monitored because of the high risk for lethal complications due to the anatomical connectivity of potential spaces. Many patients with odontogenic infections are hospitalized because of the risk of deep neck space infection (1). Odontogenic infections can be classed as either acute inflammation dominated by neutrophils or chronic inflammation characterized by mononuclear inflammatory cells (2). Symptoms such as fever, fatigue, and anorexia have been associated with the acute phase of odontogenic inflammation. Elevated high sensitivity C-reactive protein (hs-CRP) and erythrocyte sedimentation rate (ESR) have been widely used as markers of inflammatory reactions in order to estimate the presence and severity of diseases.

Platelets, as another part of the natural immune system, can be elevated in response to “acute phase reaction” during the inflammation process. In cases of active infection or inflammation (apart from acute sepsis), there is an increase in platelet count, but a decrease in MPV (3). There have been a number of reports dealing with platelet indices such as MPV which has clinical indications in various conditions such as active inflammatory diseases (4,5).

Recently, the N/L ratio has been introduced into clinical practice to evaluate systemic inflammation (6) and this ratio has been investigated to predict in a number of diseases (7-9) including cardiovascular mortality, pulmonary arterial hypertension (10), survival in malignancies, schizophrenia and hearing loss. These studies have shown that the N/L ratio is likely to be higher when compared with healthy subjects.

To the best of our knowledge, there is no study investigating the N/L ratio or MPV in odontogenic infection in the literature. Therefore, the aim of this study was to assess the value of pretreatment N/L ratio and MPV and the relation between these markers with prognostic factors such as the length of hospital stay, antibiotic doses in terms of recovery time and disease progression in patients with severe dental infection.

## Material and Methods

A cohort of 100 patients with severe dental infection who were treated in Erciyes University, Faculty of Dentistry, Oral and Maxillofacial Hospital between January 2014 and August 2014 was retrospectively examined. Approval from the local Ethics Committee was obtained prior to commencing, and the study was carried out according to the principles of the Helsinki Declaration.

The exclusion criteria were as follows: autoimmune diseases, severe systemic disease, epilepsy, hypertension, cardiac disease, hepatic or renal failure, alcohol or substance addiction, pregnancy, vitamin or fish oil intake, smoking, obesity (BMI 30 kg/m2), and concomitant drug use for any reason.

The blood tests were performed on admission to the hospital before dental treatment. Tripotassium EDTA-based anticoagulated blood samples were drawn and stored at 4°C and assessed by a Sysmex K-1000 auto analyzer within 30 minutes of sampling. Hemoglobin, hematocrit, platelets, white blood cell (WBC), differential counts (neutrophil (N), lymphocyte (L), eosinophil, basophil, and monocyte) and percentages were determined using a blood counter ADVIA 2120 Hematology System (Siemens AG, Eschborn, Germany).

The patients were divided into 2 groups according to their length of hospital stay. In group 1, the hospital stay was ‘’one day or less’’ and in group 2, the length of hospital stay was ‘’more than one day’’. The age, gender and laboratory findings in whole blood count (WBC, N, L, N/L, MPV, hemoglobin, platelet ) and inflammation markers (hs-CRP, ESR), antibiotic doses, operative procedure (drainage, extraction), anesthesia protocol and presence of fever were recorded according to these two groups ( Table 1).

**Table 1:**
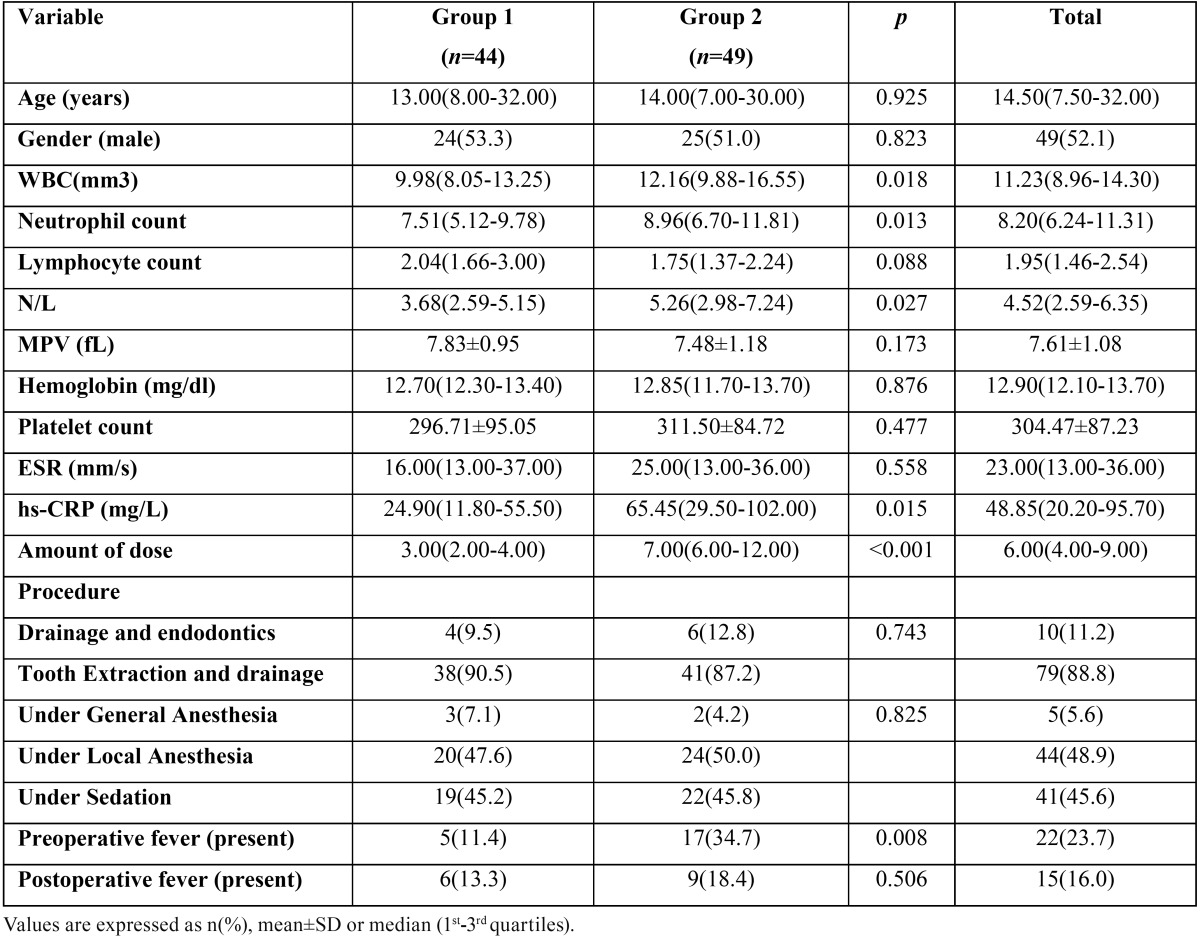
Patient characteristics.

The N/L ratio and MPV were measured in all patients. The number of preoperative antibiotic doses and postoperative doses and total doses were recorded. The correlation between preoperative fever, preoperative antibiotic doses, postoperative antibiotic doses, total antibiotic doses and hospital stay with N/L ratio and MPV were analyzed in all patients ( Table 2).

**Table 2:**
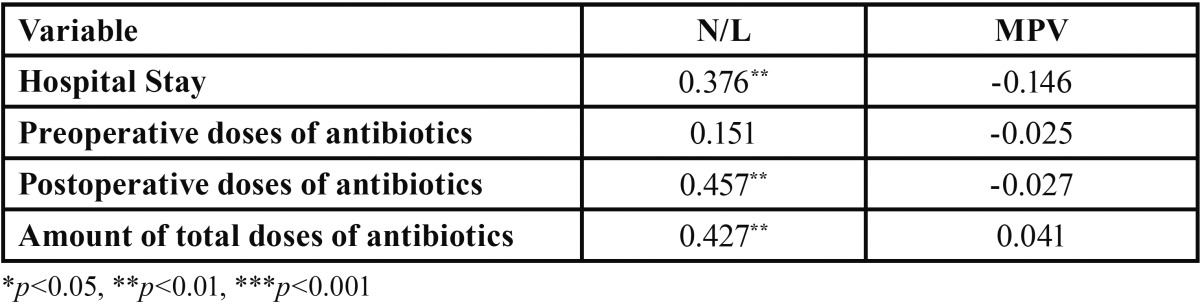
Spearman correlation coefficients for N/L, MPV and hospital stay, preoperative /postoperative and total amount of antibiotic doses.

**Figure 1:**
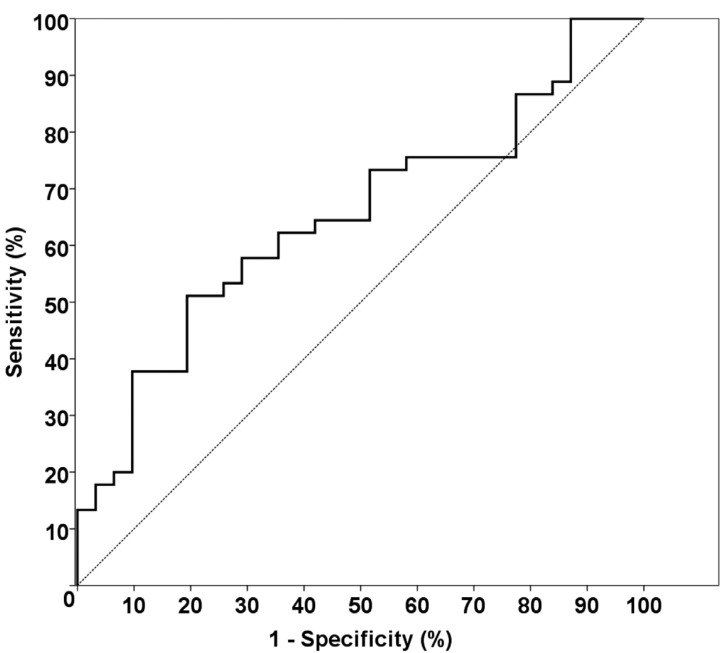
A ROC curve indicating the predictive performance of N/L on hospital stay, postoperative dose of antibiotics and total antibiotic dose.

ROC analysis was performed for identifying a cut-off value ( Table 3, Fig. 1). Moreover, patients were divided into 2 groups according to the presence of fever (group 1: fever positive (+), group 2: fever negative (-)). A temperature of 380C degrees or more was defined as positive. N/L ratio and MPV were compared between preoperative and postoperative fevers in these groups.

**Table 3:**
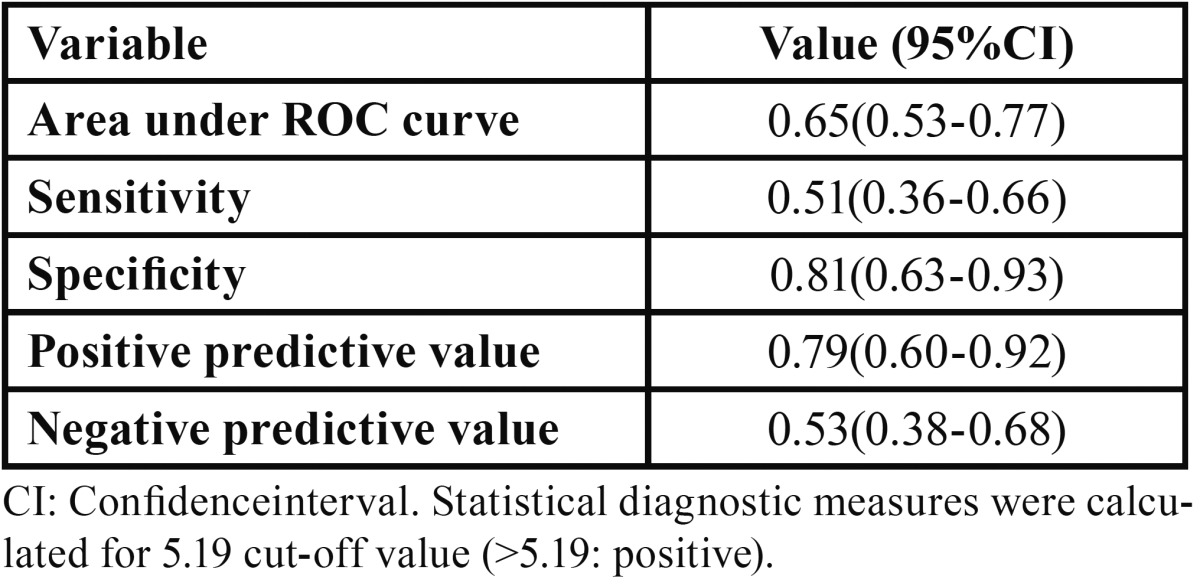
Statistical diagnostic measures of N/L in identifying hospital stay, postoperative dose of antibiotics and total antibiotic dose.

-Statistical methods

Histogram and q-q plots were examined and the Shapiro-Wilk’s test was performed to assess data normality. The Levene test was used to test variance homogenetiy. To compare the differences between groups a two-sided independent samples t test or Mann-Whitney U test was applied for continuous variables, while Pearson chi-square analysis or Fisher’s exact test was applied for categorical variables. The Spearman test was used for correlation analysis. Moreover, receiver operating characteristics curve analysis was applied to assess the predictive effect of N/L on hospital stay, postoperative dose of antibiotics and total antibiotic dose. The area under the ROC curve was calculated with 95% confidence interval. The Youden index was used to identify the optimal cut-off value. Sensitivity, specificity, as well as positive and negative predictive values were calculated with 95% confidence intervals based on the identified cut-off value. Analyses were performed using R 3.1.1 (www.r-project.org). A p value less than 5% was considered as statistically significant.

## Results

The median age was 14.50 (7.50-32.00) and 49 (52.1%) of the patients were male. Seven patients were excluded because of the absence of some postoperative blood markers. The main reason for hospitalization of patients was submandibular abscess due to permanent mandibular molar teeth (41.93%) and wisdom teeth (27.95%). Spearman correlation coefficients for N/L, MPV and hospital stay, postoperative dose of antibiotic and total antibiotic dose were obtained ( Table 2). There were positive and statistically significant correlations between N/L ratio and prolonged hospital stay and postoperative antibiotic doses and total antibiotic doses. However, there was no correlation between MPV and any of these parameters ( Table 2) .

The optimum cut–off level for the N/L ratio was detected 5.19 according to ROC analysis (sensitivity: 51, specificity: 81) ( Table 3, Fig. 1). Forty four patients were included in group 1 and 49 patient were in group 2 according to hospital stay. The patients in group 2 had a 5.19 and higher N/L ratio. In this group, the hospital stay was more than 1 day. The total antibiotic doses were 3.00 (2.00-4.00) in group 1, however it was found as 7.00 (6.00-12.00) in group 2.

When patients were divided into groups according to the presence of fever, a correlation between preoperative fever and N/L was detected ( Table 4).

**Table 4:**
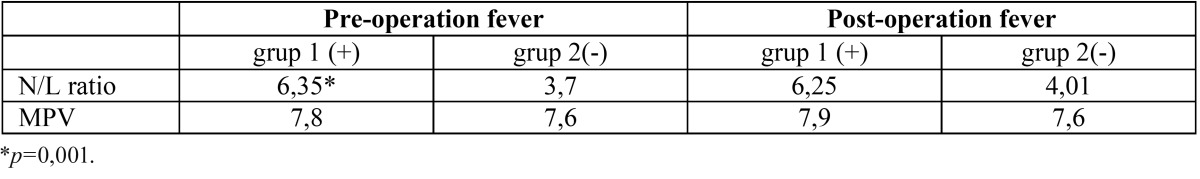
N/L ratio and preoperative fever correlation.

## Discussion

To the best of our knowledge, this is the first study to investigate the N/L ratio and MPV levels in odontogenic infections. The most important finding of our study was that the N/L ratio was associated with hospital stay and antibiotic doses in patients with odontogenic infection. The N/L ratio can easily be calculated by the ratio of neutrophils to lymphocytes in peripheral blood and that correlates with clinical status. Calculation of the N/L ratio is very simple, cheap and valuable when compared with the other inflammatory cytokines including IL-6, IL-1b, and TNF-a (11).

Odontogenic infections constitute an important part of maxillofacial surgeons’ and dentists’ clinical work. There is a high risk for lethal complications due to the anatomical connectivity of potential spaces in infections. The main reasons for hospitalization are pain, fever, and dysphagia (12-14). Early surgical drainage, hospitalization and antimicrobial treatment may be required. Clinical evaluation of the patient is of great importance to establish the appropriate therapy as soon as possible. Length of hospital stay varies from 1 to 23 days in odontogenic infections (15,16). Although conventional measures such as WBC count and ESR values are valuable in determining the clinical status of the patient at testing time, the predictability of these markers is limited in odontogenic infections (17). Sharma *et al.*, reported that hs-CRP can be an effective marker for determining severity of infection, efficacy of treatment regime and length of hospital stay for patients with fascial space infections of odontogenic origin (18). Hs-CRP is one of the acute phase reaction proteins, which is synthesized from the liver and increases during inflammatory reactions. However hs-CRP response is not agent-specific and can be identified as low for viral infections and high for acute bacterial infections (19). Leukocytosis and neutrophilia may occur with infectious diseases. The N/L ratio can also be a useful parameter to distinguish bacterial infections from viral ones (19,20). To date, the consensus diagnostic cut-off ranges of MPV and N/L ratio have not been established for the evaluation of severe dental infection.

This is the first study to investigate the N/L ratio in odontogenic infections. The N/L ratio was associated with hospital stay and antibiotic doses in patients with odontogenic infection. We found that the optimum cut–off level for the N/L ratio was 5.19 according to ROC analysis. If the N/L ratio of the patient was 5.19 or higher, more than one day of hospital stay was detected. Therefore this level could be useful for the prediction of hospital stay. According to the correlation between preoperative fever and N/L, there was an expected result that confirmed the clinical status of patients. A correlation between preoperative fever and N/L was detected.

Elevated inflammatory biomarkers such as hs-CRP and the recently identified N/L ratio were shown to be associated with prognosis in human cancers. In the maxillofacial region very limited study has been conducted on evaluation the of N/L ratio. Fang et al, reported elevated N/L ratio in a group of who were identified by means of hs-CRP to be patients at high risk of recurrence and shorter survival in oral squamous cell carcinoma. Incorporating the N/L ratio into the hs-CRP level therefore has significant potential as a biomarker for risk stratification in oral squamous cell carcinoma (21). Perisadinis *et al.*, reported that a high pretreatment N/L ratio was a significant independent predictor of shorter survival in patients with oral cancer receiving preoperative chemoradiotherapy. Moreover, there is a need to examine the N/L ratio in future studies in order to evaluate it in the maxillofacial region. An inflammatory process has been suggested to play a role in the etiopathogenesis of osteoporosis. Bone healing is delayed in the absence of lymphocytes (22). Öztürk ZA *et al.*, reported a higher N/L ratio in osteoporotic patients. They reported that there was a correlation between the N/L ratio and osteoporosis score (23). There may be a correlation between the N/L ratio and dental implant failure in the silent healing period.

Platelets are also one of the cellular parts of the immune system and change during infectious and inflammatory processes. The MPV, which is one of the hemogram parameters is also affected by many inflammatory conditions (24). Infection and inflammatory reactions usually lead to a decrease in MPV that suggests a reactive thrombocytosis (24,25). However, according to our study, MPV is not associated with the prognosis of patients with odontogenic infections. More studies are needed to determine conclusive findings.

We suggest the novel possibility that the N/L ratio may be a useful parameter for evaluating the prognosis of patients with severe dental infection. The N/L ratio may be considered as a prognostic marker for recovery time of patients. According to our study results, MPV cannot be used as a marker for odontogenic infections. This is the first study in which the N/L ratio was investigated in odontogenic infection patients and reveals that if the N/L ratio is more than 5.19, the patients may need a higher dose of antibiotics and stay more than 1 day in hospital for the treatment of odontogenic infection. On the other hand, more research is required to elucidate the role. Elevated N/L ratio predicts prolonged hospital stay and higher doses of antibiotics. Characteristically, the N/L ratio is an inexpensive and simple parameter that can be obtained by using an automatic hematology analyzer. Therefore, the N/L ratio may be further investigated for its ability to determine the clinical impact of progression in patients with other inflammatory diseases in the oral and maxillofacial region.
